# Interlaboratory comparison of an intestinal triple culture to confirm transferability and reproducibility

**DOI:** 10.1007/s44164-022-00025-w

**Published:** 2022-06-13

**Authors:** Angela A. M. Kämpfer, Ume-Kulsoom Shah, Shui L. Chu, Mathias Busch, Veronika Büttner, Ruiwen He, Barbara Rothen-Rutishauser, Roel P. F. Schins, Gareth J. Jenkins

**Affiliations:** 1 IUF – Leibniz Research Institute for Environmental Medicine, Düsseldorf, Germany; 2 School of Medicine, Faculty of Medicine, Health and Life Science, Swansea, Wales UK; 3 Adolphe Merkle Institute, University of Fribourg, Chemin des Verdiers 4, 1700 Fribourg, Switzerland

**Keywords:** Reproducibility, Interlaboratory comparison, Enterotoxicity, Diclofenac, New approach methodologies

## Abstract

The development and improvement of advanced intestinal in vitro models has received increasing attention in recent years. While the availability of relevant in vitro models is pivotal to advance the replacement and reduction of animal use in research, their robustness is a crucial determinant for intra- and interlaboratory reproducibility. We have developed a standard protocol to build a triple culture model combining two types of human intestinal epithelial cells (Caco-2, HT29-MTX-E12) and macrophages (THP-1), which was tested for transferability and reproducibility between three laboratories. The epithelial tissue barrier development and triple culture stability were investigated as well as the models’ responses to the non-steroidal anti-inflammatory drug diclofenac in terms of barrier integrity, cytotoxicity, and cytokine release. The results of two partner laboratories were compared to previously established benchmark results and quality criteria. For the epithelial co-cultures, the results were overall highly comparable between the laboratories. The addition of THP-1 cells resulted in increased variability and reduced reproducibility. While good correlation was achieved in several endpoints, others showed substantial response differences between the laboratories. Some variations may be addressed with training or demonstrations, whereas others might be related to fundamental differences in the cell lines introduced during routine cell culture and maintenance. Our results underline the importance of interlaboratory transfer studies using standardised experimental procedures, including defined quality criteria and benchmarks, as well as of training when newly establishing complex in vitro models in laboratories.

**Supplementary Information:**

The online version contains supplementary material available at 10.1007/s44164-022-00025-w.

## Introduction

The intestine is an important exposure route and target for potentially harmful compounds with countless studies aiming to elucidate their toxicity and kinetics in the organ. In the context of reducing and replacing animal testing, the development of new approach methodologies (NAMs), including in vitro models of the intestine, has been encouraged. While robust, time-saving, and cost-effective monoculture cell systems remain the backbone of toxicity testing, increasingly complex models have been presented, with gut-on-a-chip and stem cell–based organoids being the most advanced to date [1, 2]. While these sophisticated models largely remain at the proof-of-concept stage, transwell cultures with cells growing on permeable inserts—including co-cultures combining multiple cell lines—have become a standard in toxicity studies, albeit with some recognised limitations [3, 4].

Notwithstanding the developments and achievements in toxicology, both in vitro research and in vivo research struggle with a reproducibility crisis—the inability to reproduce the outcomes of others or even one’s own results [5, 6]. This lack of reproducibility causes substantial losses in material and time resources as well as unnecessary suffering and death of animals. Reproducibility is a multifactorial challenge spanning the availability of methodological information, the quality and application of research materials to protocol adherence, reporting bias, and as of yet “unknown unknowns” [5, 7]. While we assume the variability can be contained and reduced with the alignment of material origin and experimental set-up, e.g. by using standard operating procedures (SOP) [8], even sourcing cells and chemicals from the same stocks was demonstrated to be insufficient to prevent differences between laboratories [9]. The availability of the same tools and materials to different operators may still produce different outcomes as their application and overall protocol adherence can vary greatly. This includes cell culture, e.g. cell line origin, passage number, and culture maintenance, the use of consumables and reagents, as well as differences in treatment strategies, e.g. seeding densities, exposure media, and different equipment used for endpoint analysis.

While reproducibility is already problematic with monocultures [10, 11], the introduction of complex in vitro models multiplies the challenges. Co-cultures raise the effort of cell maintenance and cell line authenticity; complex in vitro models are typically long-term cultures requiring several weeks of repeated handling, which increases the risk of damage and contamination. Co-cultures may need specific culture conditions to accommodate the individual cell lines, while the model set-up might require particular handling of the components, following an elaborate succession of steps, and stringent observation of time lines.

One goal of the EU H2020 project PATROLS was to assess innovative, effective, and robust techniques to predict potential human hazards of engineered nanomaterials, which has resulted in the development of various in vitro models of the lung [12], liver [13], and intestine [14]. As one way to confirm the robustness of the developed models and the quality of the supporting materials, interlaboratory comparisons were conducted. The intestinal model used here combines co-cultures of differentiated Caco-2 and HT29-MTX-E12 cells with differentiated THP-1 cells. Its reproducibility was tested in two “naïve” laboratories of the project consortium by investigating the epithelial barrier formation, barrier integrity in presence of THP-1 cells, as well as the response to diclofenac—a non-steroidal anti-inflammatory drug used as a positive control. Diclofenac is associated with considerable gastrointestinal side effects, which are independent of metabolite formation [15, 16]. Small intestinal injury by diclofenac enhances pro-inflammatory cytokines and chemokines including interleukin (IL)8 [17], which is a crucial mediator for the recruitment of immune cells [18]. Apart from its role in inflammation, IL8 can also be an indicator for the THP-1 cell response to phorbol 12-myristate 13-acetate (PMA) [19] and was therefore used as a marker for the triple culture’s reproducibility.

## Materials and methods

### Background

The triple culture used in this study was previously described and tested for application in nanosafety studies [14]. The methotrexate-adapted HT29-MTX cell line was included in this model as mucus-producing goblet-like cell [4], with the E12 sub-clone having been identified as especially suitable for barrier formation studies and characterised by a high mucus production [20]. A seeding ratio of 9:1 Caco-2 and HT29-MTX-E12 cells, respectively, was used as it was previously identified to result in ideal barrier properties [21], and is representative of the physiological proportion of goblet cells in the human small intestinal epithelium [22]. As “host” of the model, IUF (Lab I) prepared and distributed the SOP (available at https://www.patrols-h2020.eu/publications/sops/SOP-library-pdfs/4102PATROLSSOP_Final_modifforhandbook_IUF4.1.pdf?m=1652794497&), detailing the cell culture of Caco-2, HT29-MTX-E12, and THP-1 cells as well as the set-up of the epithelial co-culture and triple culture, to the two participating “naïve” laboratories, hereinafter “Lab II” (Adolphe Merkle Institute) and “Lab III” (Swansea University). The SOP contains information on the expected characteristics of the model, benchmark values for characterisation endpoints, and quality criteria. It was accompanied by information on the exposure to diclofenac sodium salt and a video SOP demonstrating the triple culture set-up, transwell and insert handling, and sampling. No hands-on training was provided, and no materials or cryopreserved cell stocks were distributed.

In Lab II and Lab III, a person not previously acquainted with these specific cell systems conducted the experiments. In Lab I, three individual operators (OP 1–3) generated characterisation data for the epithelial co-cultures and triple cultures to provide information on intralaboratory comparability. At the time of data generation, OP2 and OP3 had 4 to 8 weeks of working experience with the co- and triple cultures. However, they received hands-on training and close supervision throughout the data generation.

The cell culture plastics (culture flasks, pipet tips, tubes) and foetal bovine serum (FBS) were not standardised between the partners unless specifically stated. The most important material differences are summarised in Table 1.

**Table 1:** Interlaboratory differences in material supply

	Lab I	Lab II	Lab III
FBS	Sigma, F7524	Gibco Life Technologies, 10,270–106	Gibco Life Technologies, 10,270–106
Culture flasks	Greiner Bio-One, 658,175	Milian, 90,076 (T75); Milian, 90,026 (T25)	Greiner Bio-One
ELISA kit	R&D DuoSet; DY208, as described in [23]	R&D systems, DY208	R&D systems, DY208
LDH assay	As described in [23]	Roche, 11,644,793,001	As described in [23]
Ohmmeter	World Precision Instruments, EVOM	Millicell, ERS-2	Millicell, ERS-2
Plate reader	Thermo Scientific, Multiskan Go	Bio-Rad Benchmark Plus	BMG Labtech, POLARstar Omega
Bright field microscope	Zeiss Axiophot	Motic AE2000, Moticam BTU 10	Zeiss AXIOVERT 40C
Fluorescence microscope	Zeiss Axio Imager M2	Leica TCS SP5	Zeiss LSM710

### Cell culture

Caco-2, HT29-MTX-E12, and THP-1 cells were all sourced and cultured according to the information detailed by Kämpfer et al. [14]. All cell lines were thawed and maintained for 3 passages before experimental use. Caco-2 and HT29-MTX-E12 cells were used for a maximum of 30 passages, THP-1 cells for a maximum of 15 passages.

### Transwell cultures

Caco-2 and HT29-MTX-E12 cells were seeded in a 9:1 ratio on Falcon 12-well transwell inserts (1-µm pore size, PET) and maintained for 21 to 22 days as described by Kämpfer et al. [14] to establish the epithelial co-culture. To obtain the triple cultures, THP-1 cells were differentiated with 100 nM PMA (Sigma, P1585) for 24 h and added to the basolateral side of the transwell system.

### Exposure to diclofenac sodium salt

Diclofenac was obtained from Sigma-Aldrich (D6899). A 100 mM stock solution was prepared in H_2_O. As the compound’s solubility in H_2_O is limited at room temperature, the stock solution was warmed up to 37 °C in a preheated water bath, for the salt to completely dissolve. The stock solution was diluted 1:50 in pre-warmed culture medium to obtain the exposure concentration of 2 mM, of which 500 µL was added to the apical compartment of the transwells. The triple cultures were maintained for 24 h before exposure to diclofenac started. Epithelial co-cultures and triple cultures were exposed for 24 h. Untreated epithelial co-cultures were included as controls.

### Barrier integrity

Throughout transwell culture maintenance, triple culture, and diclofenac exposure, the barrier integrity was measured as transepithelial electrical resistance (TEER). The participating laboratories used different Volt-Ohm-Meter with chopstick electrode (Table 1).

### Lactate dehydrogenase (LDH) assay

Cytotoxicity was analysed by quantification of LDH activity. Lab I and Lab III used the protocol described by Kämpfer et al. [23]. Lab II used the Cytotoxicity Detection Kit (LDH) from Roche (Table 1) according to the manufacturer’s instructions. For the analysis, supernatants were collected after 48 h of triple culture and 24 h of exposure, and analysed immediately without dilution. To account for variability in optical density between the different protocols, the results are presented as fold-change to the respective control.

### Interleukin (IL)8 release

The release of IL8 was quantified in undiluted apical and basolateral supernatants after 48 h of culture and 24 h of exposure. Lab I followed the protocol described in Kämpfer et al. [23], Lab II and Lab III used the same duo-set antibodies (DY208, RnD Systems) and corresponding protocol.

### Stainings

After 48-h triple culture, the transwell filters were fixed in 4% paraformaldehyde and stained for (i) tight junction network (zonula occludens (ZO-)1), cytoskeleton (F-actin), and nuclei, and (ii) neutral and acidic mucus. A detailed description of the protocols is included in the Supplementary Information, Section 1.1. The bright field and fluorescence microscopes used by the individual partners are summarised in Table 1.

### Statistics

All experiments were performed at least in triplicate with two or three biological replicates. The quality of the data was assessed according to the eligibility criteria defined in the SOP. The data was analysed with Microsoft Excel. Visualisation and statistical analysis were performed with GraphPad Prism 9. The applied statistical tests are specified in the figure legends.

## Results

### Barrier development and barrier integrity of triple cultures

**Fig. 1:**
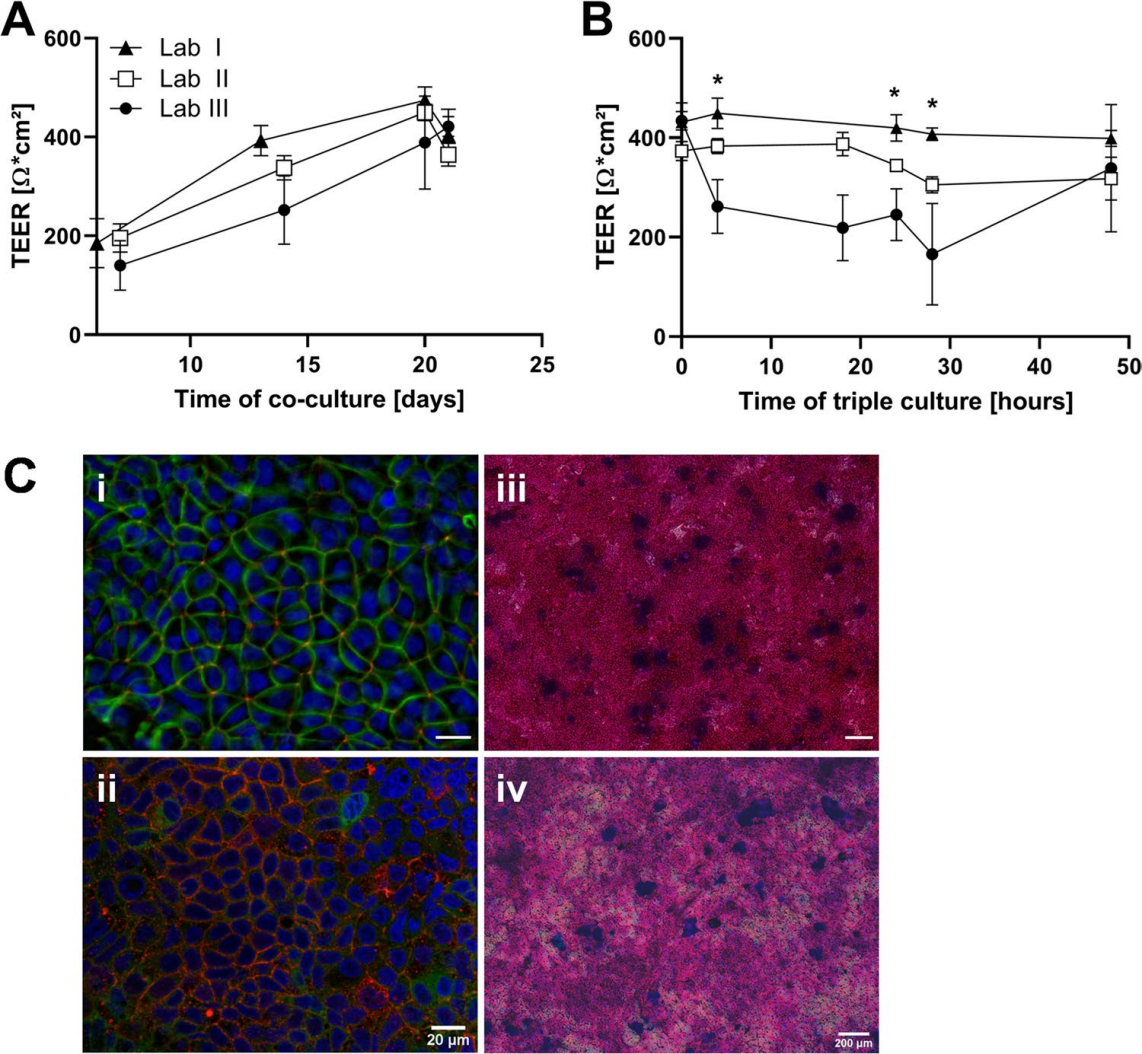
Interlaboratory comparison of (**A**) barrier formation of epithelial co-cultures measured as TEER over 21 days, and of (**B**) barrier integrity over 48-h triple culture after addition of THP-1 cells at day 21 (t_0_) (mean ± SD, *N* ≥ 3, *p ≤ 0.05 by one-way ANOVA and post hoc Tukey’s test). (**C**) Representative images of epithelial co-cultures after 48-h triple culture with THP-1 cells of Lab I (i, iii) and Lab II (ii, iv) for barrier morphology (nuclei, blue; ZO-1, red; F-actin, green) and mucus distribution (pink, neutral mucus; blue, acidic mucus) (immunofluorescence images: 40 × magnification, scale bar = 20 µm; bright field images: 10 × magnification, scale bar = 200 µm)

The TEER was measured during co-culture maintenance and throughout the triple culture to assess barrier formation and barrier integrity in presence of PMA-differentiated THP-1 cells, respectively (Fig. 1).

The barrier formation of epithelial co-cultures was overall comparable between the laboratories (Fig. 1A). Following heterogeneity over the first 14 days, no significant differences were measured among the laboratories at day 21, reaching TEER levels between 370 and 420 Ω•cm^2^.

Throughout the THP-1 triple culture (Fig. 1B), the barrier integrity remained constant in Lab I and Lab II. Lab III observed a strong reduction in TEER after 4 h of addition of the macrophages, reaching statistical significance compared to Lab I (t_4_, t_24_, and t_28_) and Lab II (t_4_ and t_24_). After 48 h, the TEER recovered to 338 ± 128 Ω•cm^2^. The TEER values of the epithelial co-cultures were more homogenous and stable over the assessment period (Figure S1).

All three participating laboratories have stained fixed transwell cultures after 48 h triple culture with THP-1 cells. Due to quality limitations, representative images of Lab I and Lab II are shown in Fig. 1C. Staining of the tight junction network and cytoskeleton (Fig. 1C) showed an intact, dense epithelial monolayer after 48-h triple culture with THP-1 cells in both Lab I (i) and Lab II (ii). The PAS reaction/alcian blue staining resulted in an overall strong pink colour reaction on the epithelial co-cultures showing the presence of neutral mucus. Islands of blue staining indicate the presence of acidic mucus, which is typically released by the HT29-MTX-E12 cells. While the PAS reaction resulted in a less homogenous colour formation in Lab II (iv), the distribution of acidic mucus appeared comparable to Lab I (iii).

An additional intralaboratory comparison between three operators of Lab I demonstrated a highly uniform development of the epithelial barrier (Figure S2A) as well as minimal TEER variability throughout 48 h triple culture with differentiated THP-1 cells (Figure S2B).

### Barrier integrity in response to diclofenac exposure

**Fig. 2:**
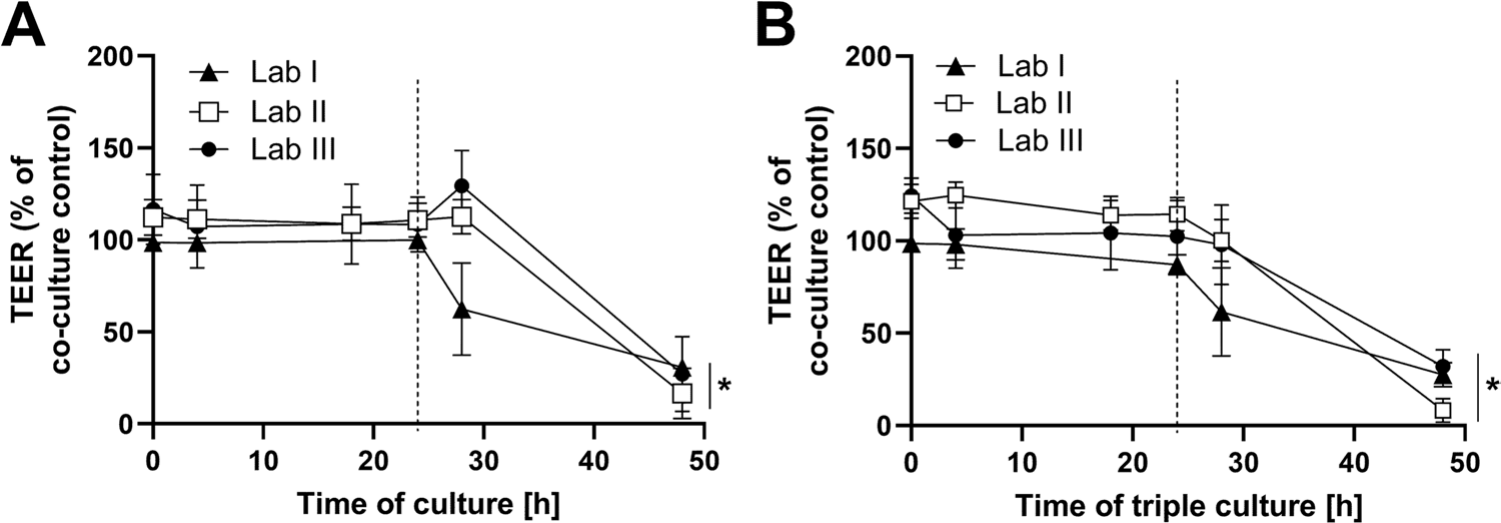
Interlaboratory comparison of the effects of diclofenac on barrier integrity measured as TEER in (**A**) epithelial co-culture and (**B**) triple culture. The epithelial co-culture and triple cultures were established and maintained for 24 h before apical treatment with 2 mM diclofenac started (indicated by a dotted line). The exposures were maintained for 24 h (mean ± SD, *N* = 3; *p ≤ 0.05 compared to corresponding unexposed control by *t*-test)

The epithelial co-cultures and triple cultures were maintained for 24 h before apical exposure to 2 mM diclofenac was started and continued for 24 h. The barrier integrity measurements are summarised in Fig. 2. In epithelial co-cultures (Fig. 2A), all laboratories measured a significant decrease in barrier integrity after 48 h to between 16 and 30% of the unexposed control.

In triple cultures (Fig. 2B), no significant differences in TEER were observed between the laboratories before the diclofenac exposure. In all laboratories, the barrier integrity was significantly reduced after 24-h exposure to diclofenac, reaching levels between 8 and 32% of the unexposed epithelial co-culture.

### Cytotoxicity following diclofenac exposure

**Fig. 3:**
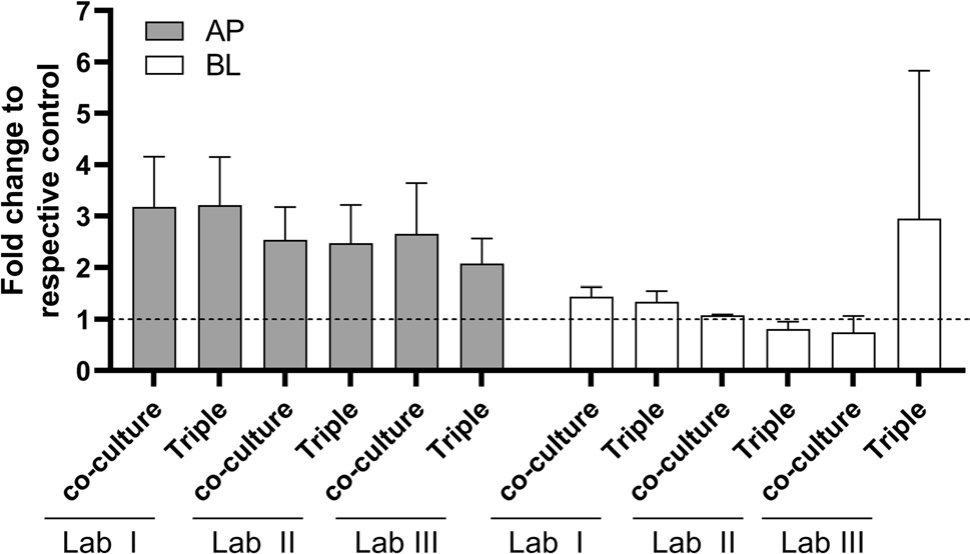
Interlaboratory comparison of LDH release in apical (grey bars) and basolateral (white bars) supernatants of epithelial co-cultures and triple cultures after 24-h exposure to diclofenac (mean ± SD, *N* ≥ 3)

All laboratories reported a noticeable increase in apical LDH activity in diclofenac-exposed epithelial co-cultures and triple cultures (Fig. 3). Increase in LDH activity was quantified in basolateral supernatants, except for exposed triple cultures in Lab III. Due to the considerable standard deviation, however, this might result from sample collection or analysis.

### IL8 release

**Fig. 4:**
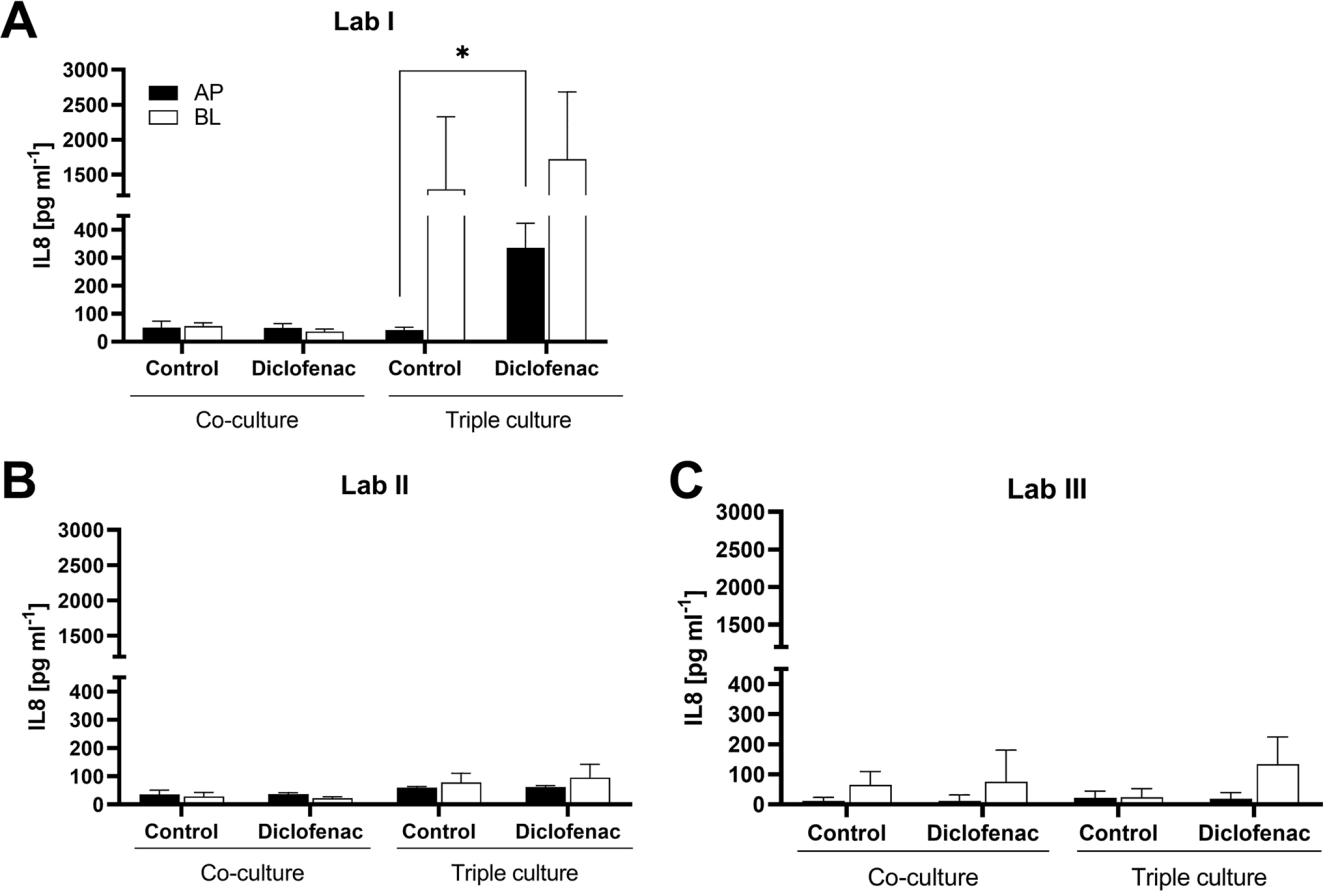
Interlaboratory comparison of IL8 release in apical (black bars) and basolateral (white bars) supernatants of epithelial co-cultures and triple cultures after 24-h exposure to diclofenac (mean ± SD, *N* ≥ 3; *p ≤ 0.05 against corresponding control by *t*-test)

IL8 was quantified to assess the PMA differentiation of THP-1 cells as well as the response of the cell systems to diclofenac (Fig. 4). The THP-1 cells reportedly responded well to the PMA differentiation as indicated by adherence and morphological changes (Figure S3).

In Lab I, IL8 was detected at low levels (55 ± 22 pg mL^−1^) in both apical and basolateral supernatants of the epithelial co-cultures, and did not increase following exposure to diclofenac (Fig. 4A). In triple cultures, the basolateral IL8 content was strongly increased. After diclofenac exposure, the IL8 release was increased in both apical (*p* = 0.0089) and basolateral supernatants to ~ 340 and 1700 pg mL^−1^, respectively.

In the epithelial co-cultures, Lab II and Lab III quantified similar IL8 concentrations, and did not observe an increase in response to diclofenac (Fig. 4B, C). In the triple culture, however, their results differed substantially from Lab I: In control cultures, the basolateral IL8 content remained low (78 and 23 pg mL^−1^); neither the apical nor the basolateral IL8 content increased in response to diclofenac.

## Discussion

In line with previous reports [24], diclofenac induced significant adverse effects in the investigated intestinal in vitro models. The exposure concentration of 2 mM was considerably higher than plasma concentrations following the oral intake of diclofenac sodium salt [25, 26].

### Complexity reduces reproducibility

The results demonstrated an overall comparable barrier development and cytotoxicity response to diclofenac of the epithelial co-cultures between the laboratories. However, when THP-1 cells were included, the results became more heterogeneous and reproducibility within and between the laboratories decreased. Nonetheless, the strong cytotoxic effect of diclofenac was detected by all partners.

The assessment of TEER offers an ideal parameter for monitoring epithelial tissue barrier development and integrity in response to stresses. Even though barrier integrity and the measurement can be affected by temperature, cell passage, medium composition, positioning of the electrode, etc. [27], TEER has been recommended for inter- and intralaboratory comparison [28]. Despite Caco-2 cells being regarded as uncomplicated and low-maintenance, large variations in TEER, differentiation parameters, and other cell characteristics have been described between commonly used (and often misclassified) sub-clones, passage number/age, and culture conditions [28–31]. As the three participating laboratories used different Ohmmeters and starting passages of the cell lines, identical TEER values were not expected. Considering these differences, the homogeneity in barrier development and diclofenac-induced barrier disruption was striking. This supports the assumption that the original source of the cell lines and the culture conditions, including cell seeding density and culture medium composition, are crucial factors for the reproducibility, which is in line with the interlaboratory comparison results summarised by Zucco et al. [28].

### THP-1 cells introduced variability

The most substantial difference was detected in IL8 release, as only Lab I quantified significant levels of the chemokine in control and exposed triple cultures. Other studies have reported long-term stabilisation of IL8 mRNA and time and dose-dependent increase in background levels of secreted IL8 following differentiation of THP-1 cells with PMA [19, 32]. Already during the development of the predecessor model, established in a different laboratory and using another batch of THP-1 cells, the PMA-dependent increase in background IL8 was observed, while cell line authenticity and absence of *Mycoplasma* were established [23].

PMA is photosensitive; therefore, storage conditions and light exposure might negatively affect its impact on THP-1 cells. However, it is unlikely that the PMA differentiation was hindered altogether, as both partner labs reported adherence and morphological changes (Figure S3), common indicators for THP-1 differentiation [33]. Nevertheless, the differences among the laboratories remain unexplained. Variations in the PMA exposure, e.g. regarding concentrations, exposure lengths, or resting periods, were demonstrated to significantly affect THP-1 identity [23, 33, 34] as well as the cells’ cytokine release, phagocytic capacity, and generation of reactive oxygen species (ROS) [19, 33]. Adherence to the protocol, therefore, is essential for the reproducibility of THP-1 differentiation and the triple culture. Apart from the differentiation protocol also the culture conditions can affect THP-1 cell response to PMA, as demonstrated by Aldo et al. [35]. Any differences in cell culture prior to the PMA differentiation, even before the cryopreservation of the stock, might have influenced the cells. To investigate and, ultimately, exclude these factors, the comparison would have to be conducted with THP-1 cells from one source (i.e. one of the partner labs) or a simultaneously ordered fresh stock from one supplier.

The absence of background IL8 might cause the lack of apical IL8 in diclofenac-exposed triple cultures of Lab II and Lab III. Due to the heavily disrupted epithelial barrier, apical and basolateral supernatants could have mixed. This would explain both the increased apical levels in Lab I, where IL8 translocated from the basolateral side, and the unchanged levels in Labs II and III. However, in Lab I, diclofenac exposure increased the IL8 concentration by 40% compared to the control, which suggests a de novo chemokine formation. Caco-2 and HT29 cells are able to produce and release IL8 upon stimulation with cytokines, LPS (HT29 only), or ROS [36, 37], albeit at a lower capacity than immune cells [38]. Often, the IL8 release is paralleled by cell death or vice versa [39, 40], but as no enhanced release was detected in epithelial co-cultures, the toxicity mechanism of diclofenac presumably does not induce an IL8 stress response in the epithelial cells.

Instead, the IL8 induction may be related to the presence of THP-1 cells, their differentiation, or activation status. Depending on the origin and type of stimulation, Caco-2 cells can secrete IL8 predominantly to the apical, basolateral, or to both sides equally. For instance, basolateral exposure to tumour necrosis factor alpha (TNFα) induced both apical and basolateral IL8 secretion, while apical exposure only enhanced apical release [41]. While TNFα is absent from supernatants of the triple culture [14, 42], interleukin 1 beta (IL1β), another potent inducer of IL8, was previously detected at sufficiently high concentrations [24, 43]. Epithelial cell reaction to diclofenac may differ depending on the presence of THP-1 cells and the constitutively secreted levels of stimuli like IL8 and IL1β, which can affect the magnitude of a subsequent cytokine response [38].

## Conclusion

Having tested one substance at a single concentration, this study is merely a proof-of-concept investigation. However, its outcomes underline the importance of interlaboratory trials to identify methodological limitations and shortcomings of instruction materials, while supporting the conclusions from previous interlaboratory comparisons.

Our observations suggest that respecting a minimum set of parameters can be sufficient to reliably reproduce more complex in vitro systems, as long as the cell lines involved do not require additional differentiation treatments. For the here tested epithelial co-culture characteristics and endpoints, it was not necessary to align every detail in cell culture (e.g. FBS), passage number, or instrumentation (Ohmmeter). However, THP-1 variability remains challenging and cannot be accounted for simply by sourcing the cells from the same supplier. The establishment of a (complex) model as well as assessing the reproducibility between laboratories is greatly facilitated by the availability of detailed instructions, defined characterisation benchmarks, and quality criteria, while hands-on training stands out as a crucial factor. To investigate responses among laboratories, control substances such as diclofenac can be advantageous over suspended materials like nanoparticles. When live demonstrations are not possible, the provision of visual materials like video SOPs can facilitate explaining complex procedures and experimental handling. The model characterisation should be focused on easily measurable endpoints of limited variability, while quality criteria ideally comprise upper and lower bounds, where applicable. Sufficient time and resources need to be reserved to implement and characterise a complex model prior to its experimental application.

## Supplementary Information

Below is the link to the electronic supplementary material.

Supplementary file1 (DOCX 1360 KB)

## Data Availability

The datasets used and analysed during the current study are available from the corresponding author on reasonable request.
