# Supplementary material for: Interlaboratory comparison of an intestinal triple culture to confirm transferability and reproducibility
Source: In Vitro Model. 2022 Jun 13;2(3-4):89–97. doi: 10.1007/s44164-022-00025-w (PMC11756443; doi:10.1007/s44164-022-00025-w)
Supplement: Supplementary file 1 — Supplementary file1 (DOCX 1360 KB) [file 44164_2022_25_MOESM1_ESM.docx]

**Supplementary Information**

**Interlaboratory comparison of an intestinal triple culture to confirm transferability and reproducibility**

Angela A.M. Kämpfer^1^, Ume-Kulsoom Shah^2^, Shui L. Chu^3^, Mathias Busch^1^, Veronika Büttner^1^, Ruiwen He^3^, Barbara Rothen-Rutishauser^3^, Roel P.F. Schins^1^, Gareth J. Jenkins^2^

^1^ IUF – Leibniz Research Institute for Environmental Medicine, Düsseldorf, Germany

^2^ School of medicine, Faculty of Medicine, Health and Life Science, Swansea, Wales, UK

^3^ Adolphe Merkle Institute, University of Fribourg, Chemin des Verdiers 4, 1700 Fribourg, Switzerland

**1 Materials & methods**

**1.1 Immunocytochemical and –histological staining of transwell filters**

Barrier integrity

Samples were fixed in 4 % paraformaldehyde (PFA, 20 min, room temperature), washed with PBS, permeabilized (0.1% Triton X-100, 5 min), and blocked against unspecific binding (3% BSA/PBS, 30 min). The specimens were incubated with ZO-1 (2.5 μg mL^− 1^) antibody (Thermo Fisher, 617300) for 1h at room temperature. After washing, the samples were incubated with AlexaFluor594-conjugated secondary antibody (1:300) (Thermo Fisher, A11037), AlexaFluor488-conjugated phalloidin reconstituted in DMSO (1:1000) (Thermo Fisher, A12379), and Hoechst 33342 (0.5 mg mL^-1^) in 1% BSA/PBS for 30 min at 37°C protected from light. The washed samples were mounted onto standard microscopy slides using Prolong Gold anti-fade mounting medium (Thermo Fisher, P36934). IUF assembled re-assembled the individual channels of its images using ImageJ.

Mucus identification

PFA-fixed samples were treated with 3% acetic acid for 3 min and 1% Alcian blue in 3% acetic acid (Sigma Aldrich, 101647) for 30 min. Subsequently, washed specimens were incubated with periodic acid (Sigma Aldrich, P7875) (1% in H_2_O) for 10 min. Subsequently, Schiff’s reagent (Merck, 1.09033.0500) was added and the samples incubated for 15 min protected from light. Following washing with sulphite water (3x 2 min) and demineralised water (1x 10 min), the samples were mounted onto microscopy glass slides using Prolong Gold Antifade reagent and analysed by light microscopy within 5 days. IUF has enhanced brightness equally over the whole of its image using ImageJ. No quantitative analyses was performed on the images.

2 Supplementary Figures

**Fig. S1** Barrier integrity measured as TEER over an assessment period of 48h from day 21 to day 23. (mean ± SD, N≥3)

**Fig. S2** Epithelial barrier formation during 21 days cultivation (A) and barrier integrity for triple cultures during 48 hours (B). Intralaboratory comparison of the three operators of Lab I (mean ± SD, N=3)

**Fig. S3** Bright field microscopy images of THP-1 cells after 24h PMA differentiation from (A) laboratory I, (B) laboratory II and (C) laboratory III.
